# Reduction of Radiation Dosage in Visualization of Paranasal Sinuses in Daily Routine

**DOI:** 10.1155/2017/3104736

**Published:** 2017-01-31

**Authors:** Christian Güldner, Isabell Diogo, Julia Leicht, Magis Mandapathil, Thomas Wilhelm, Afshin Teymoortash, Evelyn Jahns

**Affiliations:** ^1^Department of ENT, Head and Neck Surgery, UKGM, Marburg, Germany; ^2^Department of Otolaryngology, Head/Neck & Facial Plastic Surgery, Sana Kliniken Leipziger Land, Borna, Germany

## Abstract

*Background*. Preoperative imaging of the nose and paranasal sinus is standard in otorhinolaryngology. Previous studies on phantoms demonstrated the potential for dose reduction of cone beam computed tomography (CBCT) by varying the application parameters.* Methodology.* Based on previous studies, the standard protocol of paranasal sinus imaging by CBCT was altered. One hundred and fifty examinations using the old protocol (01/2010–01/2011, high dosage) and 150 examinations using the new protocol (09/2012–09/2013, low dosage) were evaluated and compared for the visibility of 17 anatomical structures, the Lund-Mackay Score, and technical parameters.* Results.* Alteration of the protocol resulted in a significant reduction in dosage (6.64 mGy versus 2.88 mGy). Both groups showed the same amount of pathology (Lund-Mackay Score: 4.95 ± 3.79 versus 5.26 ± 5.77; *p* = 0.558). There was a significant better visibility of the anatomical structures (all visible = 1, nothing visible = 4) (results: 1.25 versus 1.17; *p* = 0.001) in the low-dosage group.* Conclusion.* Despite a significant reduction in the applied dosage, reliable visualization of the bony anatomy of the anterior skull base is possible by CBCT. This demonstrates the need for the discussion of the required clinical imaging quality.

## 1. Introduction

Diseases of the nose and paranasal sinuses are important from the medical as well as from the socioeconomic point of view [1]. Besides patient history and clinical examination, radiological visualization is an important diagnostic tool [2]. The options include conventional plane radiography, ultrasound, magnetic resonance imaging (MRI), computed tomography (CT, MSCT), and cone beam computed tomography (CBCT). To date, no correlation between anatomical variants and the extent of disease has been shown, so the main indication for imaging is the preoperative visualization of risk structures and the prevention of complications [3]. According to current guidelines, preoperative imaging has to be at least in two planes [1, 2, 4]. Classic plane radiography can be used to search for focus diseases but seems to play no important role at all [1, 2, 4]. The domain of MRI is the visualization of the soft tissue and should be used in cases with central or orbital complications or in the diagnosis of malignant diseases [1, 2, 4]. In daily routine, CT is the workhorse. Studies over the last 10 years have demonstrated the power of CBCT in precise visualization of the bony structures of the nose and paranasal sinuses [5–8].

Given the use of X-rays in CT or CBCT, the question of dose reduction should be addressed in every procedure. A discussion of the imaging quality required should also take place. It could be shown that the previous mentioned fact realizes a successful reduction of dosage [9, 10]. As a result of this, CBCT and low-dose CT come to the dosage region of plain radiography with significant higher content of information [11]. Previous studies were performed mainly on cadavers or phantoms. Therefore, the question of transferability into daily routine and potential limitations in humans remains unanswered. Based on previous studies performed by our group [10], the standard protocol of CBCT examinations of the paranasal sinuses and anterior skull base was changed to a new low-dose protocol. This allowed the comparison of a high-dose and low-dose protocol in a clinically relevant number of patients, which was the main goal of the current study.

## 2. Material and Methods

All parts and analysis of the study were permitted by the Ethical Committee of the University of Marburg, Germany. According to the local guidelines for research on retrospective human data, no specific approval was necessary. To ensure a representative result, 340 datasets on the nose and paranasal sinuses were randomly extracted retrospectively from the CBCT database by taking every third examination for further analysis. All recordings were initially indicated and performed in collaboration with the Department of Otorhinolaryngology and Neuroradiology in patients with suspicion of chronic rhinosinusitis or simple traumatology of the midface. One hundred and seventy examinations were from the period 01/2010 to 01/2011 (old standard protocol: high dosage, group 1) and 170 examinations were from the period 09/2012 to 09/2013 (new standard protocol: low dosage, group 2). Due to an incomplete field of view, five datasets from group 1 and 19 datasets from group 2 had to be excluded. Thus, 316 examinations were available for further analysis (group 1: *N* = 165; group 2: *N* = 151). In terms of technical parameters, the tube voltage, tube current, rotation angle of the tube, the field of view, and the applied dosage, given by the device (computed tomography dose index, CTDI), were recorded. All examinations were performed using the CBCT device produced by Morita (Accu-I-Tomo F17, Morita, Kyoto, Japan).

**Figure 1:**
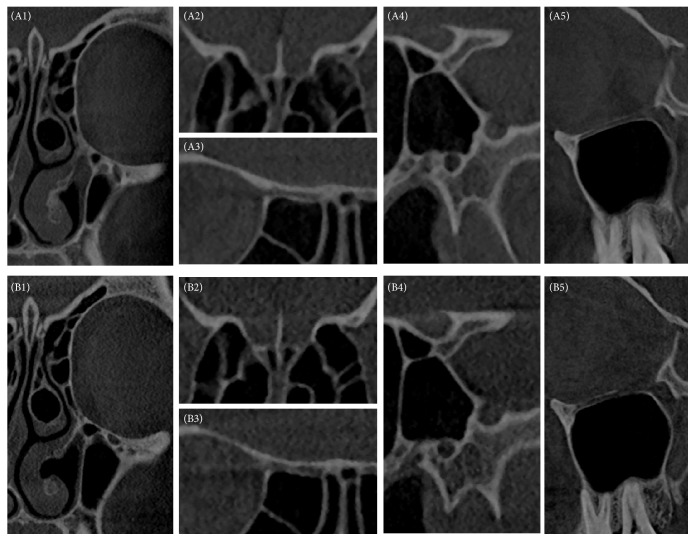
Example of patient examined with both the old (A, group 1) and the new (B, group 2) protocol. Typical images of the anatomical structures are presented to give an impression of the influence of dose reduction (1, os turbinale of inferior turbinate and lamina papyracea; 2, olfactory fossa and anterior ethmoidal artery; 3, posterior ethmoidal artery; 4, optical nerve canal, maxillary neve canal, and vidian nerve canal; 5, infraorbital nerve canal).

The following anatomical parameters were evaluated using a four-point Likert scale (1 = excellent visibility, 2 = good visibility, 3 = poor visibility, and 4 = not visible): (1) lateral wall of maxillary sinus; (2) uncinate process; (3) bony part of the inferior turbinate; (4) bony canal of the infraorbital nerve; (5) lamina papyracea at the point of the uncinate process; (6) bony canal of the anterior ethmoidal artery; (7) lamina papyracea at the point of the anterior ethmoidal artery; (8) lateral wall of the olfactory fossa; (9) cribriform plate; (10) bony canal of the posterior ethmoidal artery; (11) bony canal of optical nerve; (12) bony canal of vidian nerve; (13) bony canal of maxillary nerve; (14) bony canal of internal carotid artery; (15) nasolacrimal duct; (16) posterior wall of the frontal sinus. Examples are given in Figure 1. To ensure comparability, the mean of all anatomical structures was calculated.

Additional, the Lund-Mackay Score was recorded to analyse the extent of disease [12]. All reviews of the anatomic structures and amount of pathology (Lund-Mackay Score) were performed by one examiner who was blinded to the applied dosage and the adjustments of the single examination.

Statistical analyses were performed using SPSS 22.0 (SAS Statistics, Cary, NC, USA). Between-group comparisons were made using the Chi-Square Test. Nonscaled parameters were compared using *t*-test for independent values.

## 3. Results

Overall, 316 datasets were evaluated. There were no significant differences between groups in either age (41.9 ± 17.3 years versus 37.7 ± 17.3 years; *p* = 0.482) or gender distribution (group 1: female = 41% versus group 2: female = 66%).

All technical parameters of the X-ray tube favoured the new low-dose standard protocol. Tube current (5.28 mA versus 3.96; *p* = 0.000) and tube voltage (88.24 kV versus 85.51 kV; *p* = 0.000) were significantly lower in group 2. With regard to the rotation angle of the tube, in group 1, 100% of the examinations were performed using the 360° mode and in group 2 100% of the examinations used the 180° mode. Despite a bigger field of view (FOV) in group 2 (group 1: 100% examinations with a 10 × 10 cm FOV versus group 2: 100% examinations with a 14 × 10 cm FOV), in combination with the application parameters, the applied dosage was significantly lower in group 2 (group 1: 6.64 mGy versus group 2: 2.88 mGy; *p* = 0.000). These results are summarized in Table 1.

The amount of pathology, measured using the Lund-Mackay Score, did not differ significantly between groups but showed a tendency for higher values in group 2 (group 1: 4.95 ± 3.79 versus group: 5.26 ± 5.77; *p* = 0.558) (Table 1).

Figure 1 gives an impression of the consequence of dose reduction. Both images are from one patient who received an examination using the old (group 1) and new (group 2) standard protocol. Therefore, a direct comparison of the visibility of the anatomical structures is possible.

A detailed analysis of the individual anatomical structures is given in Table 2. Only the uncinate process and the cribriform plate were significantly more visible in group 1. The differences in the uncinate process might be a consequence of the differences in the amount of pathology between groups. Regarding the cribriform plate, group 1 showed better results for excellent and good visibility but worse results for poorly visible and not visible in comparison to group 2 (see Table 2). The bony canal of the internal carotid artery was significantly more visible in group 2.

In determination of the mean value of the evaluation of the single anatomic parameters, a significantly better visualization was possible in group 2 (group 1: 1.25 versus group 2: 1.17; *p* = 0.001).

To exclude the influence of the parameters which seem to be visible excellent or well independent from the applied dosage (lamina papyracea at the level of anterior ethmoidal artery, canal of the optical nerve, canal of the vidian nerve, canal of the maxillary nerve, nasolacrimal duct, and posterior wall of the frontal sinus), a selected mean of the remaining parameters was calculated. Again, a significant advantage was detected for group 2 (group 1: 1.35 versus group 2: 1.26; *p* = 0.033) (Table 1).

## 4. Discussion

Given the frequent use of X-ray-dependent examinations in the diagnosis of diseases of the nose and paranasal sinuses, the available devices, adjustments, and examination parameters should be under continuous improvement. In terms of imaging optimization, there is a conflict between dose reduction and imaging quality. So far, protocols with reduced tube current-time product and tube voltage have been developed in CT [13, 14]. Furthermore, protocols with lens-shields or tilted gantry realize a dose reduction in daily routine [15]. In CBCT, as well as tube voltage and current, variation of the rotation angle of the tube is possible in most devices. When using the 180° mode, the tube rotates and irradiates only at the back of the head, which results in significantly lower irradiation of the lens and a reduction of the effective dosage [16]. Even in regular paranasal sinus protocols, comparing high class devices of CT and CBCT, CBCT has about half of the applied dosage [17]. The disadvantage of many studies performed to date is their focus or use of phantoms or cadavers [9]. Therefore, the comparability of these results to daily routine is not known. Based on previous papers focusing on imaging quality and dose reduction [9, 10], the standard protocols for the available CBCT device were changed in our institution. Afterwards, relevant patient groups from the old and new protocol were compared. In terms of the cohort statistics (age, sex, and the level of pathology), the two groups were comparable. A significant reduction in dosage (6.64 mGy versus 2.88 mGy) without any impact on the imaging quality was apparent (group 1: 1.25 versus group 2: 1.17; *p* = 0.001). Only the uncinate process and the cribriform plate were more visible in the high dosage group. This may reflect slight differences in the level of pathology. In group 2, there were slightly more pathologies of the anterior ethmoid, which makes it difficult to differentiate the uncinate process from the obstructed anterior ethmoid. Even when comparing a selected mean score (only parameters that showed dose-dependent visibility), there was no disadvantage for group 2 (group 1: 1.35 versus group 2: 1.26; *p* = 0.033).

The limitations of CBCT should not be forgotten. The focus of CBCT is high-contrast imaging and this does not allow any differentiation of soft tissue. Therefore, in suspicious central or orbital complications, CT or preferably MRI should be used. But in respect of the correct indication, CBCT has advantages over CT and should be considered as an alternative [18]. In patients requiring focus on the bony anatomy of the anterior or lateral skull base, CBCT is a meaningful diagnostic device [19]. De Cock et al. presented a study showing the limitations of CBCT in patients with extended chronic polypoid rhinosinusitis. In their conclusion, the small restriction in visibility was compensated by the significantly lower irradiation [17]. In the same way, Leiva-Salinas et al. concluded in their study that despite higher noise and a lower signal-to-noise ratio, the lower dosage applied in CBCT in comparison to MSCT favours the former and allows reliable examination of the paranasal sinuses [20].

Besides the fact of the discussion of the clinical needed imaging quality, the optimization of technical parameters results in a dose reduction. For example, the implementation of specific filters in postimaging processing produces better visibility with lower irradiation. Another issue of interest is the diagnostic workup. Pletcher et al. demonstrated that the diagnostic power of primary registered coronary planes is equivalent to that of 0.625 mm axial reconstructed planes and even better than that of 1 mm axial reconstructed planes [21].

The main limitations of the present study are the analysis by only one observer and the use of only one CBCT device and the relatively low level of pathologies in the patient cohort. But this reflects our daily routine. Further studies could and should focus on this topic in more detail.

In conclusion, even with a significant reduction of the applied dosage, reliable visualization of the bony anatomy of the anterior skull base was possible using cone beam CT in a relevant patient cohort. This shows the need and potential for an intensive discussion of the conflict between the clinically required imaging quality and the dosage applied by radiologists/neuroradiologists together with ORL surgeons and physicians.

## Figures and Tables

**Table 1:** Overview of the technical parameters of the X-ray tube, the applied dosage, the Lund-Mackay Score, and the mean evaluation score for all structures and the selected group of anatomical parameters for the two dosage groups.

	High dosage	Low dosage	*p* value
Age in years	41.9 ± 17.3	37.7 ± 17.3	n.s.
Sex (female : male)	67 : 98	100 : 51	n.s.
Tube current in mA	5.28 ± 1.26	3.96 ± 0.49	0.000
Tube voltage in mA	88.24 ± 3.40	85.51 ± 1.01	0.000
Rotation angle	360° (100%)	180° (100%)	0.000
Computed tomography dosage index (CTDI) in mGy	6.64 ± 0.98	2.88 ± 0.33	0.000
Field of view (diameter in cm × height in cm)	10 × 10	14 × 10	
Lund-Mackay Score	4.95 ± 3.79	5.26 ± 5.77	0.558
Mean of anatomic structures	1.25 ± 0.23	1.17 ± 0.16	0.001
Selected mean of anatomic structures	1.35 ± 0.29	1.26 ± 0.23	0.033

**Table 2:** Overview of the frequencies in percent of the evaluation of the visibility of the different anatomical structures in the two groups (high dosage versus low dosage) with the corresponding *p* values (^*∗*^values marked with an asterisk are highly significant).

	High dosage (*N* = 165)				Low dosage (*N* = 151)				*p* value
	Excellent	Well	Poor	Not evaluable	Excellent	Well	Poor	Not evaluable	
Maxillary sinus (lateral wall)	79.6	15.5	4.9	0.0	80.7	15.3	4.0	0.0	0.791
Uncinate process	66.9	18.8	13.4	0.9	67.4	11.1	14.8	6.7	0.022^*∗*^
Inferior turbinate (os turbinale)	87.8	7.0	4.3	0.9	87.0	7.3	4.3	1.3	0.917
Infraorbital nerve canal	54.5	32.4	12.7	0.3	59.3	29.3	11.3	0.0	0.682
Lamina papyracea (infundibulum)	85.8	9.1	5.2	0.0	93.7	5.0	1.3	0.0	0.119
Anterior ethmoidal artery canal	84.2	11.5	3.6	0.6	91.7	7.7	0.7	0.0	0.107
Lamina papyracea (ant. ethm. art.)	95.2	1.8	3.0	0.0	97.0	2.3	0.7	0.0	0.248
Lateral lamella olfactory fossa	86.4	10.0	3.6	0.0	93.3	3.7	3.0	0.0	0.148
Cribriform plate	94.4	27.0	21.8	1.8	73.0	18.7	8.3	0.0	0.000^*∗*^
Posterior ethmoidal artery canal	67.9	15.8	10.0	6.4	75.7	13.0	8.7	2.7	0.238
Optical nerve	93.3	4.3	2.4	0.0	98.3	1.3	0.3	0.0	0.176
Vidian nerve	94.2	2.1	3.0	0.6	97.7	2.3	0.0	0.0	0.211
Maxillary nerve	95.4	2.4	2.1	0.0	99.0	0.7	0.3	0.0	0.320
Carotid artery	83.0	12.8	3.5	0.6	97.7	1.7	0.7	0.0	0.003^*∗*^
Nasolacrimal duct	98.5	1.5	0.0	0.0	99.3	0.7	0.0	0.0	0.406
Frontal sinus	98.2	1.2	0.6	0.0	99.3	0.7	0.0	0.0	0.386
